# M30 Antagonizes Indoleamine 2,3-Dioxygenase Activation and Neurodegeneration Induced by Corticosterone in the Hippocampus

**DOI:** 10.1371/journal.pone.0166966

**Published:** 2016-11-21

**Authors:** Chun-Sing Lam, George Lim Tipoe, Johnny Kong-Ching Wong, Moussa B. H. Youdim, Man-Lung Fung

**Affiliations:** 1 School of Biomedical Sciences, The University of Hong Kong, Hong Kong, Hong Kong SAR; 2 Research Centre of Heart, Brain, Hormone & Healthy Aging, Li Ka Shing Faculty of Medicine, The University of Hong Kong, Hong Kong, Hong Kong SAR; 3 Department of Pharmacology & Pharmacy, The University of Hong Kong, Hong Kong, Hong Kong SAR; 4 Eve Topf Center for Neurodegenerative Diseases Research, Faculty of Medicine, Technion-Israel Institute of Technology, Haifa, 31096, Israel; Chiba Daigaku, JAPAN

## Abstract

Monoamine oxidases (MAO), downstream targets of glucocorticoid, maintain the turnover and homeostasis of monoamine neurotransmitters; yet, its pathophysiological role in monoamine deficiency, oxidative stress and neuroinflammation remains controversial. Protective effects of M30, a brain selective MAO inhibitor with iron-chelating antioxidant properties, have been shown in models of neurodegenerative diseases. This study aims to examine the neuroprotective mechanism of M30 against depressive-like behavior induced by corticosterone (CORT). Sprague-Dawley rats were given CORT subcutaneous injections with or without concomitant M30 administration for two weeks. CORT-treated rats exhibited depressive-like behavior with significant elevated levels of MAO activities, serotonin turnover, oxidative stress, neuroinflammation and apoptosis in the hippocampus with significant losses of synaptic proteins when compared to the control. The expression and activity of cytokine-responsive indoleamine 2,3-dioxygenase (IDO-1), a catabolic enzyme of serotonin and tryptophan, was significantly increased in the CORT-treated group with lowered levels of serotonin. Besides, CORT markedly reduced dendritic length and spine density. Remarkably, M30 administration neutralized the aberrant changes in the hippocampus and prevented the induction of depressive-like behavior induced by CORT. Our results suggest that M30 is neuroprotective against CORT-induced depression targeting elevated MAO activities that cause oxidative stress and neuroinflammation, resulting in IDO-1 activation, serotonin deficiency and neurodegeneration.

## Introduction

Major depressive disorder is a life-threatening psychological disorder highly prevalent in the worldwide population [1, 2]. Clinically, depression is closely associated with hypercortisolemia in patients, which may be involved in the atrophy and dysfunction of the hippocampus [3, 4]. This is consistent with the findings that chronic exposure to corticosterone (CORT) induces depressive-like behavior in rodents with aberrant dendritic arborization and impaired synaptic plasticity in the hippocampus [5, 6]; yet the pathophysiological mechanism of chronic CORT treatment leading to the monoamine deficiency and neurodegeneration remains controversial.

Monoamine oxidases (MAO), with two isoforms A and B, are enzymes located at the outer membrane of mitochondria that catalyze the oxidative deamination of monoamine neurotransmitters and produce hydrogen peroxide as a by-product [7]. MAO-A is mainly responsible for the deamination of serotonin, norepinphrine and dopamine, whereas MAO-B degrades phenethylamine, benzylamine and dopamine [8]. Elevated brain MAO-A activities have been reported in both living and post-mortem tissues of clinically depressed patients [9–11], which were also found to be implicative in the pathogenesis of stress-induced depressive-like behaviors in experimental animals [12, 13]. Anomalous activation of the MAO-A activity could alter the turnover and availability of monoamines resulting in serotonin deficiency, manifested as one of the major clinical observations [14]. Thus, pharmacological inhibition of MAO-A is a first-line clinical treatment for the patient [15]. Notably, MAO-A is one of the main downstream targets of glucocorticoids and potentially plays a pathophysiological role in CORT-induced depressive-like behavior. However, the mechanistic effect of blockade of MAO activities against the pathophysiological cascade of CORT-induced depressive-like behavior remains unclear. In this context, recent studies proposed a significant role of neuroinflammation in the brain of clinically depressed patients [16]. Putatively, it could induce depressive-like behavior in rodents with an activation of inflammatory cytokines-responsive indoleamine 2,3-dioxygenase (IDO-1), which is a key enzyme for the catabolism of tryptophan and serotonin, that could deplete the level of serotonin [17, 18]. In addition, the metabolites of IDO-1 have been reportedly shown to induce neuronal apoptosis and neurodegeneration as a result of IDO-1 activation [19, 20]. Yet, it remains elusive the role of neuroinflammation and IDO-1 in CORT-induced depression.

M30, 5(-N-Methyl-N-propargylaminomethyl)-8- hydroxyquinoline), is brain-permeable to the blood brain barrier and is a potent brain-selective MAO inhibitor with chemical properties of iron-chelating free radical scavengers [21]. It is composed of propargyl moiety for the MAO inhibition and the prototype of iron-chelator VK28. Experimental studies have demonstrated the beneficial effect of M30 against the pathogenic cascade of neurodegenerative processes in rodent models of Alzheimer’s or Parkinson disease, via suppressing the brain MAO activity and oxidative stress [22, 23]. Recently, M30 has also been shown to effectively alleviate the elevated level of inflammatory cytokines in a mouse model of Alzheimer’s disease [24]. However, the mechanistic effect of M30 against neuroinflammation induced by CORT remains elusive. We hypothesized that M30 is neuroprotective against CORT-induced depressive-like behavior. This study focused on the pathophysiological mechanism leading to the CORT-induced depressive-like behavior, in which oxidative stress and neuroinflammation mediated by over-activation of MAO and IDO-1 activities could significantly contribute to the serotonin deficiency and neurodegeneration.

## Materials and Methods

### Animals and experimental grouping

All experimental procedures were approved and conducted according to the Committee on the Use of Live Animals in Teaching and Research (CULATR #2522–11, 3545–15), The University of Hong Kong. The Laboratory Animal Unit of the University of Hong Kong is fully accredited by the Association for Assessment and Accreditation of Laboratory Animal Care International (AAALAC international). Adult male Sprague Dawley rats (220–250 g) were put under pathogen-free condition in an air-conditioned room at constant temperature (23±1°C) provided with water and standard diet (LabDiet, 5053 (LabDiet; St. Louis, MO, USA)) ad libitum. All animals were monitored on a daily basis for body health throughout the study. There were five experimental groups (n = 12 per group), namely non-treated control (Control), M30-treated groups (M30), corticosterone-treated group (CORT), corticosterone and M30 co-treated group (CORT + M30) and vehicle-treated group (Vehicle).

### Drug preparation and treatment

Drug M30, 5(-N-Methyl-N-propargylaminomethyl)-8- hydroxyquinoline) was kindly provided by Dr. Moussa Youdim and Dr. Lin Bin. M30 solution was freshly prepared by dissolving in saline. Corticosterone (Sigma, St Louis) was prepared daily using sesame oil as vehicle and injected subcutaneously (near the cervical region) according to the method described by Hellsten et al. [25]. A daily injection of corticosterone at a dose of 50 mg/kg for consecutive 14 days significantly elevated the levels of CORT (S1 Table) and MAO expression (S1 Fig) and also induced depressive-like behavior in rats as previously reported [6]. M30 at a dose of 5 mg/kg was intraperitoneally injected to the animals with or without CORT co- treatment for 14 days. The animals were anesthetized with halothane and then decapitated to harvest the hippocampus for experiments.

### Measurement of MAO-A and MAO-B activities

Hippocampi were homogenized in 50 mM potassium phosphate buffer (pH = 7.4) and the lysate was diluted in the reaction buffer provided by Amplex Red Monoamine Oxidase Assay Kit (Invitrogen, CA, USA). The MAO-A enzyme activity was quantified according to the manufacture protocol and was normalized to total protein content in each sample. The results were expressed as percentage of the control.

### Western Blot

Protein expressions of hippocampal tissue lysate (including cytosolic and nuclear fractions) were performed as previously described [26]. The expression of β-actin served as the internal control for whole cell lysate and cytosolic fraction protein, whereas Lamin B1 served as the internal control of nuclear fraction protein. Primary antibodies of SOD-2 (rabbit polyclonal, 1:1000), GPx-1 (goat polyclonal, 1:500), NFκB p65 (rabbit polyclonal, 1:250) and p50 (mouse monoclonal, 1:250), IκBα (mouse monoclonal, 1:500), TNF α (goat polyclonal, 1:80), IL-1β (rabbit polyclonal 1:100), IL-6 (goat polyclonal, 1:1000) and COX-2 (goat polyclonal, 1:100) were purchased from Santa Cruz Biotechnology, CA, USA; Synapsin 1 (rabbit polyclonal, 1:500) and Synaptophysin (rabbit polyclonal, 1:2000) were purchased from Novus Biologicals, USA; PSD95 (rabbit polyclonal 1:500), Cleaved Caspase 3 (rabbit polyclonal 1:500) was purchased from Cell Signaling Technology; Cleaved PARP-1 (rabbit polyclonal, 1:2000) was purchased from Bioworld Technology; IDO-1 (rabbit polyclonal, 1:250) was purchased from antibodies-online (ABIN1714836). The optical density of the bands was measured and quantified by Image J (National Institute of Health, MD, USA). The data were expressed as percentage of the control.

### Enzyme-Linked Immunosorbent Assay (ELISA)

According to manufacturers’ protocol, ELISA kits were employed to measure the levels of serotonin (5-HT) (Enzo Life Sciences), 5-HT metabolite (5-HIAA) (Elabscience), tryptophan (TRP) (LDN Labor Diagnostika Nord GmbH & Co.KG), Kynurenine (KYN) (MyBioSource) and Quinolinic acid (QUIN) (Cloud-Clone Corp.) in the hippocampal tissue lysate. The results were expressed as ng/gram wet tissue and nM respectively.

### Malondialdehyde (MDA) Assay

MDA amount of hippocampal samples was measured by Bioxytech LPO-586TM kit (OxisResearch, Portland, OR). In brief, according to manufacturer’s instruction, the reaction products were read at 586 nm followed by normalization of corresponding protein amount measured by Bio-Rad Protein Assay Kit (Bio-Rad, Hercules, CA). Standard curve was constructed with 1,1,3,3-tetraethoxypropan. The results were expressed as μmol/mg and percentage of the control.

### GSH/GSSG Ratio

Hippocampus was homogenized with 5% metaphosphoric acid followed by centrifugation at 14,000g for 15min at 4°C to obtain the supernatant. According to manufacturer’s protocol, the total amount of glutathione (GSH) and oxidized glutathione (GSSG) were determined. The amount of reduced GSH was calculated using the equation: Reduced GSH = Total glutathione–oxidized GSSG. The results were expressed as GSH/GSSG ratio.

### Golgi Staining

Golgi staining was performed to reveal morphological features of hippocampal neurons with the use of FD Rapid GolgistainTM Kit (FD Neurotechnologies, MD). According to the manufacturer’s instruction, hippocampal CA1 and CA3 pyramidal neurons are visualized for the analysis of dendritic length and spine density. Five neurons from each 100μm-thick transverse section of the hippocampus were sampled and analyzed with the use of Neurolucida software (MicroBright-Field, USA). Neurons selected for the analysis were relatively isolated and distinguished from neighboring impregnated neurons to avoid interference. The area of cell bodies, dendritic length and spine density of both apical and basal branches were quantified. The volume of cell body was calculated with the use of the mathematic equation: 4/3πr^3^. For the dendritic length and spine density, a minimum of three to five with at least one branch point were selected for counting. Visible spines along the branch segment were counted and the spine density was expressed as number per 10μm.

### Behavioral Tests

Forced swimming test (FST) was employed to examine the depressive-like behavior of the rats. The immobility time served as the valid indicator of behavioral despair according to the previous reports [27, 28]. In brief, rats were placed in a cylinder (60cm height X 25 cm diameter) containing tap water for 15-min training session to learn helplessness on the first day after 2-week CORT treatment. Rats were then put into the water again for 5 minutes and the performance was recorded on the second day. The immobility time was evaluated.

Reward-based sucrose consumption was carried out to assess the hedonic status of the rodents with the use of sucrose preference test (SPT) [29, 30]. Another independent batch of rats were individually caged and given the training session in which two bottles of 1% (wt/vol) sucrose solution for 24 hours on the first day after 2-week CORT treatment to prevent the subtle stress when applying the sucrose consumption assessment. After that, the rats were provided one bottle of water and the other contained 1% (wt/vol) sucrose solution for 24 hours. The positions of bottles were swapped in the middle of the assessment to avoid the bias towards a particular side. No food and water deprivation was applied before the test to prevent the interference with the metabolic demands of the rats. The consumption of water and sucrose solution was recorded by weighing bottles before and after the test. Sucrose consumption was presented as the percentage of the sucrose solution over the total weight of liquid consumed. The data were expressed as percentage of the control.

### Statistical Analysis

Data from each group were expressed as mean ± SEM. Statistical comparisons among groups were performed using One way ANOVA followed by Tukey’s post-hoc test for multiple comparisons. A p<0.05 was considered to be statistically significant with the use of Graphpad Prism® software (Graphpad Software Version 5.01, Inc., San Diego, USA)

## Results

### M30 antagonized CORT-induced depressive-like behaviors

**Fig 1:**
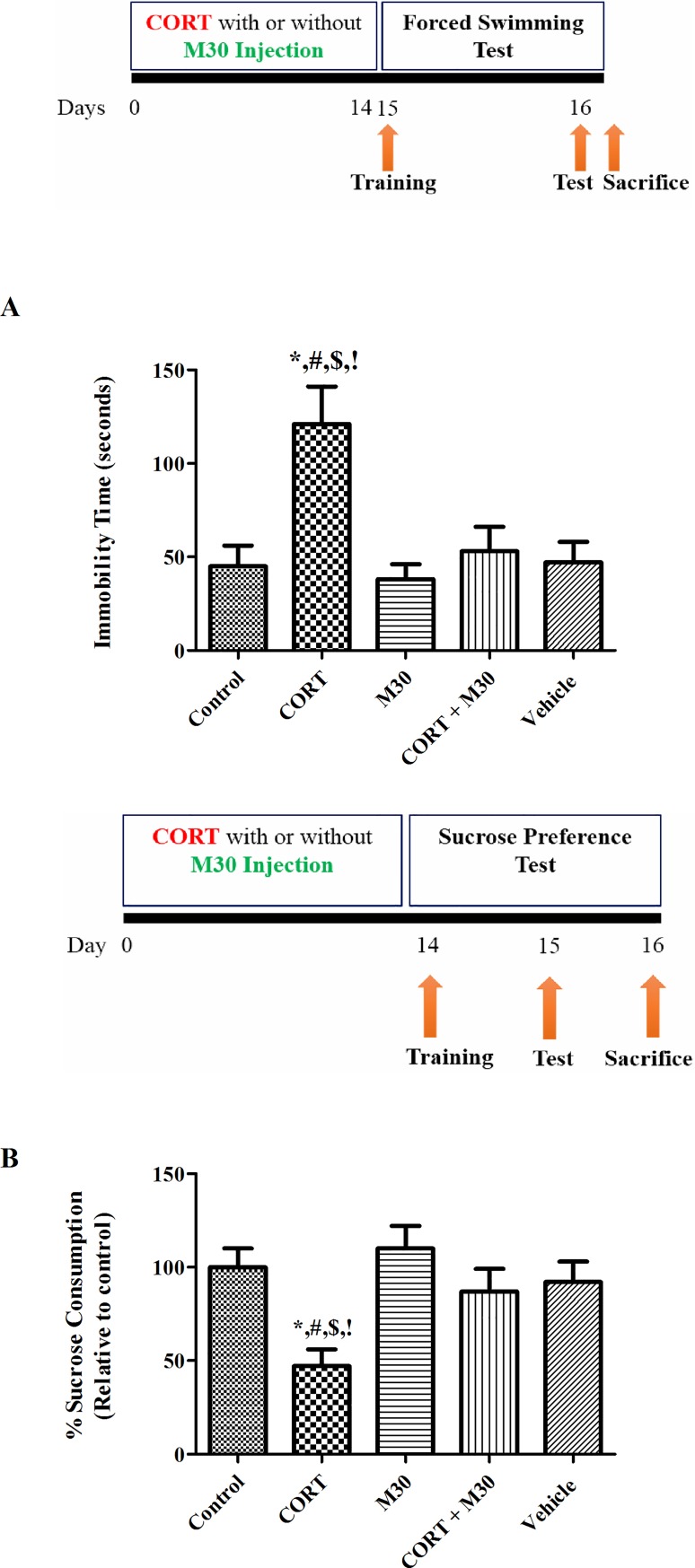
Chronic CORT treatment induced depressive-like behavior in rats, which was significantly ameliorated by the M30 administration. Panels A and B summarize the immobility time and percentage of sucrose consumption of the animals in the control, CORT-treated (CORT), M30-treated (M30), CORT and M30 co-treated (CORT+M30) or vehicle groups respectively. Data from each group were expressed as mean ± SEM (n = 12). Statistical comparisons between groups were performed using the One way Anova followed by Tukey post hoc test to detect differences in all groups. For FST, *p < 0.001 when compared with Control, ^#^p < 0.001 when compared with M30, ^$^p < 0.001 when compared with CORT + M30 groups,^!^ p < 0.001 when compared with Vehicle. For SPT, *p < 0.01 when compared with Control, ^#^p < 0.01 when compared with M30, ^$^p < 0.01 when compared with CORT + M30 groups,^!^ p < 0.05 when compared with Vehicle.

The immobility time in the forced swimming test and the percentage of sucrose consumption in the sucrose preference test were, respectively, increased and reduced significantly in the CORT-treated group when compared to the control and vehicle groups (p<0.001 for FST, p<0.01 for SPT) (n = 12, Fig 1). There were no differences between the control and the M30-treated groups with or without the CORT treatment (Fig 1). Results indicated that chronic CORT treatment induced depressive-like behavior in the rat, which was significantly prevented by the M30 administration.

### M30 mitigated CORT-induced MAO over-activation

**Fig 2:**
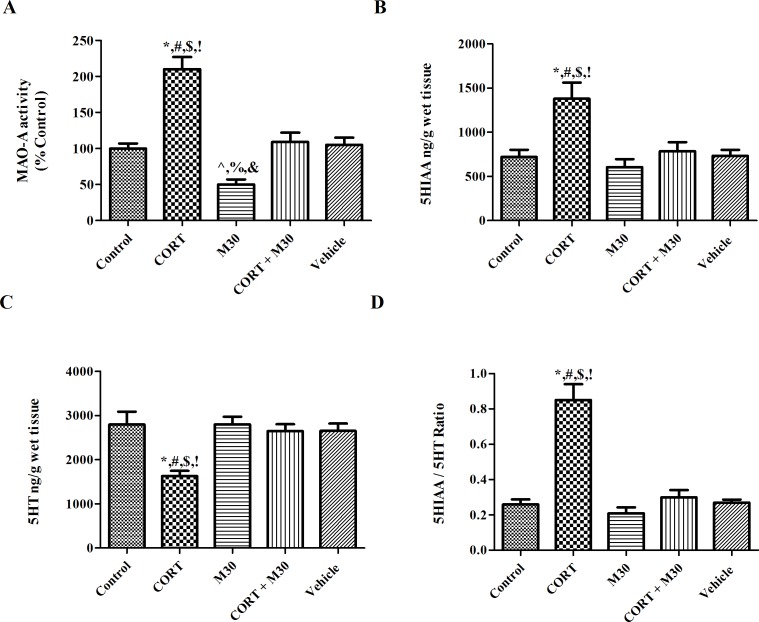
MAO-A activity and serotonin (5HT) turnover were increased in the hippocampus of CORT-treated group, which was significantly attenuated by M30. Figures A-D summarize the levels of (A) MAO-A activity, (B) 5HIAA, (C) 5HT and (D) the ratio of 5HIAA/5HT in the hippocampus of the control, CORT-treated (CORT), M30-treated (M30), CORT and M30 co-treated (CORT+M30) or vehicle groups. β-actin was the internal control. Data from each group were expressed as mean ± SEM (n = 8). Statistical comparisons between groups were performed using the One way Anova followed by Tukey post hoc test to detect differences in all groups. For MAO-A activity, *p < 0.001 when compared with Control, ^#^p < 0.001 when compared with M30, ^$^p < 0.001 when compared with CORT + M30 groups,^!^ p < 0.001 when compared with Vehicle, ^^^p < 0.01 when compared with Control, ^%^p < 0.01 when compared with CORT + M30, ^&^p < 0.01 when compared with Vehicle groups. For 5HIAA, 5HT and 5HIAA/5HT ratio, *p < 0.001 when compared with Control, ^#^p < 0.001 when compared with M30, ^$^p < 0.001 when compared with CORT + M30 groups,^!^ p < 0.001 when compared with Vehicle.

The activity of MAO-A was doubled in the CORT-treated group when compared with that of the control group (p<0.001) (n = 8), but was significantly prevented by the M30 treatment (Fig 2). Besides, there was a significant decrease in the MAO-A activity by 50% in the M30-treated group, when compared with that of the control or vehicle group (p<0.01) (Fig 2). The level of 5-HT was significantly reduced by 40% in the CORT-treated group when compared with that of the control group (p<0.001) (n = 8). The level of 5-HIAA was significantly elevated by 50% in the CORT-treated group when compared with that of the control group (p<0.001) (n = 8). In addition, the ratio of 5-HIAA/5-HT was significantly increased in the CORT-treated group when compared with the control (p<0.001). However, there was no significant change in the levels of 5-HT and 5-HIAA in the M30-treated group.

### M30 attenuated CORT-induced oxidative stress

**Fig 3:**
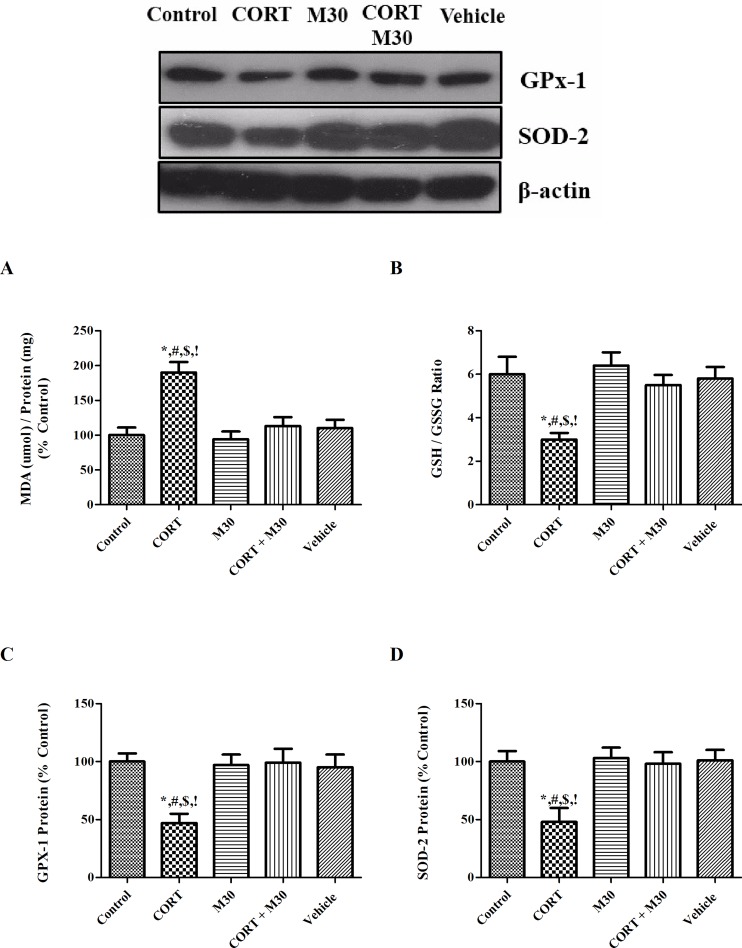
M30 attenuated MAO-induced oxidative stress. Levels of (A) MDA content, (B) GSH/GSSG ratio, and the protein expression of (C) GPx-1 and (D) SOD-2 in the hippocampus of the control, CORT-treated (CORT), M30-treated (M30), CORT and M30 co-treated (CORT+M30) or vehicle groups are summarized in the figures. β-actin was an internal control. Data from each group were expressed as mean ± SEM (n = 8). Statistical comparisons between groups were performed using the One way Anova followed by Tukey post hoc test to detect differences in all groups. For MDA, *p < 0.001 when compared with Control, ^#^p < 0.001 when compared with M30, ^$^p < 0.01 when compared with CORT + M30 groups,^!^ p < 0.001 when compared with Vehicle. For GSH/GSSG ratio, GPx-1 and SOD-2, *p < 0.01 when compared with Control, ^#^p < 0.01 when compared with M30, ^$^p < 0.05 when compared with CORT + M30 groups,^!^ p < 0.05 when compared with Vehicle.

The MDA level in the hippocampus was markedly elevated by 2 folds in the CORT-treated group when compared with that of the control group (p<0.001) (n = 8, Fig 3). The increased MDA level was significantly attenuated in the group co-treated with M30 (Fig 3). Additionally, levels of the GSH/GSSH ratio (p<0.01) and protein expressions of antioxidant enzymes GPx-1 and SOD-2 (p<0.01 for both) in the CORT-treated group were significantly reduced by 50% of the control or vehicle group, which was normalized by the M30 treatment.

### M30 suppressed neuroinflammation via redox-sensitive NFКB canonical pathway

**Fig 4:**
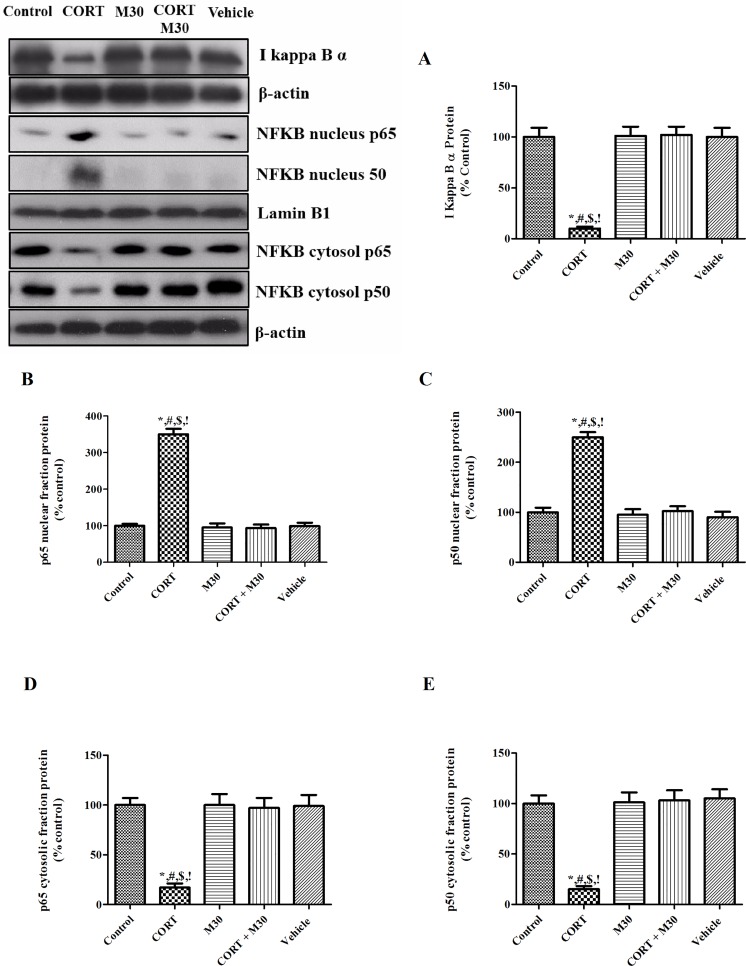
M30 prevented the degradation of redox-sensitive NFКB canonical pathway negative regulator IКBα and repressed the nucleus translocation of NFКB member p65 and p50 from cytosol. Levels of protein expression of (A) IКB α, nuclear protein expression of (B) p65 and (C) p50, and cytosolic protein expressions of (D) p65 and (E) p50 in the hippocampus of the control, CORT-treated (CORT), M30-treated (M30), CORT and M30 co-treated (CORT+M30) or vehicle groups are summarized. Lamin B1 and β-actin were an internal control of the nuclear fraction cytosolic fraction respectively. Data from each group were expressed as mean ± SEM (n = 8). Statistical comparisons between groups were performed using the One way Anova followed by Tukey post hoc test to detect differences in all groups. *p < 0.001 when compared with Control, ^#^p < 0.001 when compared with M30, ^$^p < 0.001 when compared with CORT + M30 groups,^!^ p < 0.001 when compared with Vehicle.

**Fig 5:**
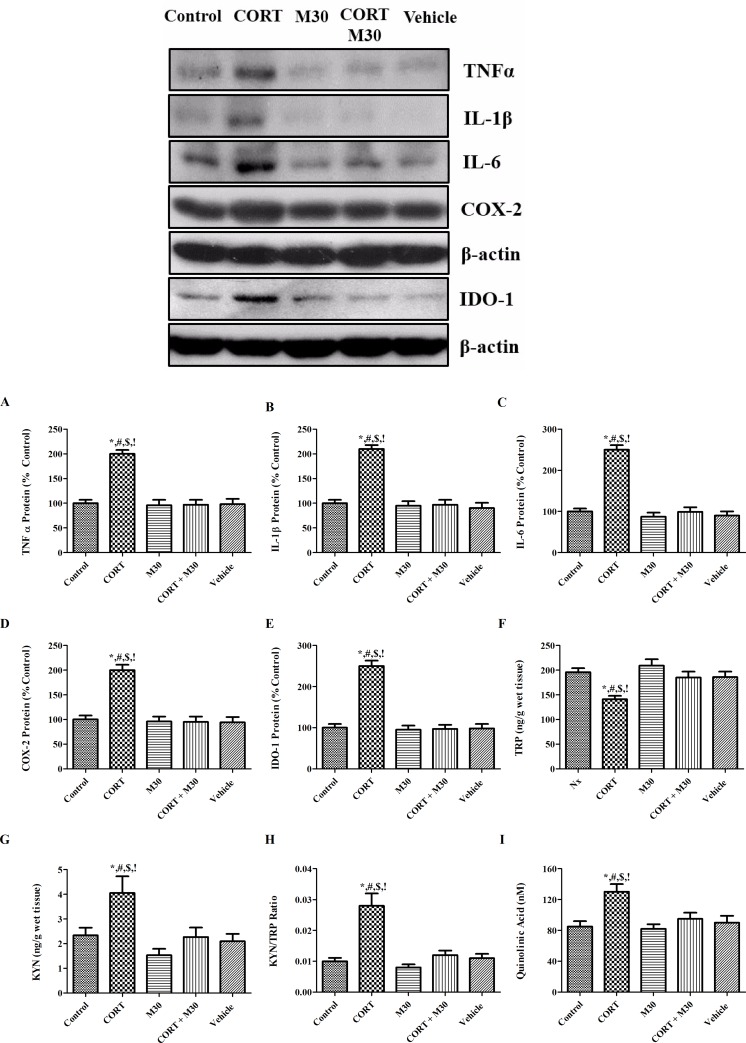
Chronic CORT treatment induced neuroinflammation and activated cytokine-responsive IDO-1 in the rat hippocampus, which was significantly mitigated by M30 administration. Levels of the protein expression (A) TNFα, (B) IL-1β, (C) IL-6, (D) COX-2, (E) IDO-1, (F) TRP, (G) KNY, (H) the ratio of KYN/TRP (IDO-1 activity) and (I) the level of QUIN in the hippocampus of the control, CORT-treated (CORT), M30-treated (M30), CORT and M30 co-treated (CORT+M30) or vehicle groups are summarized. Data from each group were expressed as mean ± SEM (n = 8). Statistical comparisons between groups were performed using the One way Anova followed by Tukey post hoc test to detect differences in all groups. For TNFα, IL-1β, IL-6 and COX-2, *p < 0.001 when compared with Control, ^#^p < 0.001 when compared with M30, ^$^p < 0.001 when compared with CORT + M30 groups,^!^ p < 0.001 when compared with Vehicle. For TRP, KYN, KYN/TRP and QUIN, *p < 0.01 when compared with Control, ^#^p < 0.01 when compared with M30, ^$^p < 0.01 when compared with CORT + M30 groups,^!^ p < 0.01 when compared with Vehicle.

The amount of total I-kappa-Bα, the negative regulator of NFКB canonical cascade was significantly decreased in the CORT-treated group when compared with the control groups (p<0.001) (n = 8, Fig 4). Consistently, the nuclear-fractional expressions of p65 and p50 were significantly augmented by 3.5 and 2.5 folds (p<0.001 for both), respectively, whereas the cytosolic fractional expressions of p65 and p50 were remarkably lowered by 80% and 90%, respectively (p<0.001 for both). The degradation of I-kappa-Bα and translocation of NFКB members p65 and p50 were significantly repressed by the M30 treatment (Fig 4). Also, the protein expressions of inflammatory cytokines TNFα, IL-1β, IL-6 and COX-2 were significantly elevated by 2–2.5 folds in the CORT-treated group when compared with that of the control or vehicle group (p<0.001 for all) (n = 8). The elevated levels of cytokine were significantly ameliorated by the M30 treatment (Fig 5).

### M30 blocked the expression and activity of IDO-1 and attenuated the level of QUIN

The protein expression of IDO-1 in the CORT-treated group was 2.5 folds higher than that of the control or vehicle group (n = 8, Fig 5). The level of TRP was reduced by 30% in the CORT-treated group when compared with that of the control (p<0.01) whereas the level of KYN was elevated by 40% in the CORT-treated group when compared with that of the control (p<0.01). The kynurenine to tryptophan (KYN/TRP) ratio was doubled in the CORT-treated group when compared with that of the control (p<0.01). IDO-1 downstream metabolite quinolinic acid was significantly elevated in the CORT-treated group when compared with that of control (p<0.01). The increased levels of IDO-1 expression, TRP, KYN, KYN/TRP ratio and QUIN were significantly attenuated in the group co-treated with M30 (Fig 5).

### M30 abrogated CORT-induced hippocampal apoptosis

**Fig 6:**
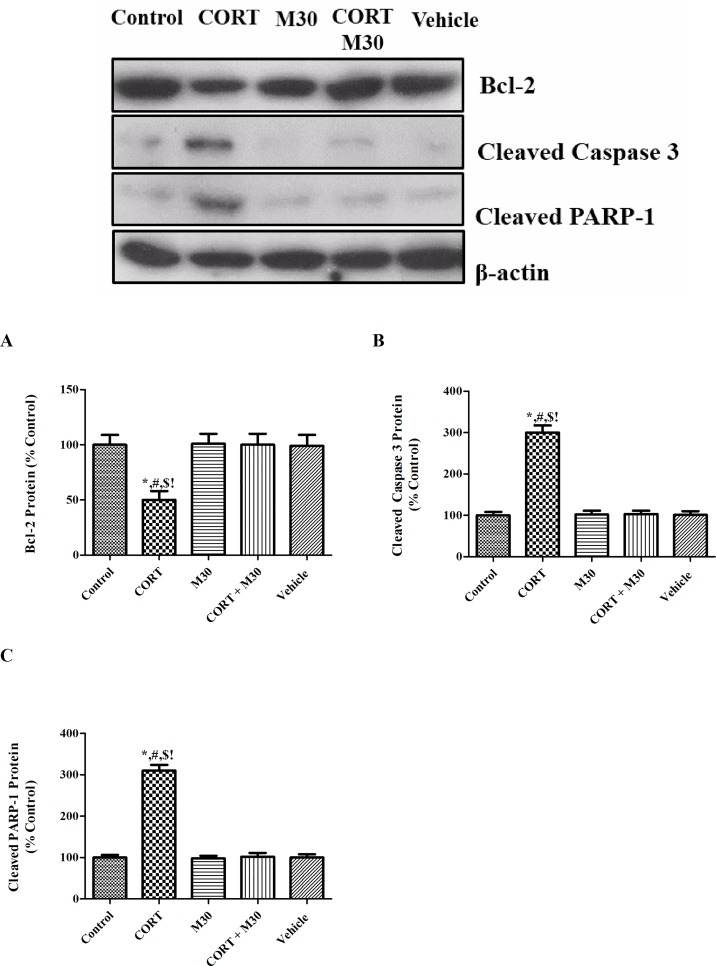
M30 abrogated CORT-induced hippocampal apoptosis. Levels of protein expression of (A) Bcl-2, (B) Cleaved Caspase 3 and (C) Cleaved PARP-1 in the hippocampus of the control, CORT-treated (CORT), M30-treated (M30), CORT and M30 co-treated (CORT+M30) or vehicle groups are summarized. β-actin was an internal control. Data from each group were expressed as mean ± SEM (n = 8). Statistical comparisons between groups were performed using the One way Anova followed by Tukey post hoc test to detect differences in all groups. For Bcl-2, *p < 0.01 when compared with Control, ^#^p < 0.01 when compared with M30, ^$^p < 0.01 when compared with CORT + M30 groups,^!^ p < 0.01 when compared with Vehicle. For cleaved caspase 3 and cleaved PARP-1, *p < 0.001 when compared with Control, ^#^p < 0.001 when compared with M30, ^$^p < 0.001 when compared with CORT + M30 groups,^!^ p < 0.001 when compared with Vehicle.

The expression level of anti-apoptotic protein Bcl-2 in the CORT-treated group was significantly less by half of that of the control group (p<0.01). In addition, levels of the apoptotic markers, cleaved-caspase-3 and cleaved-PARP-1, were increased by 3 folds in the CORT- treated group when compared with those of the control or vehicle group (p<0.001 for both) (n = 8, Fig 6). No changes were found in the M30-treated groups when comparing to the control (Fig 6).

### M30 restored CORT-induced loss of synaptic proteins

**Fig 7:**
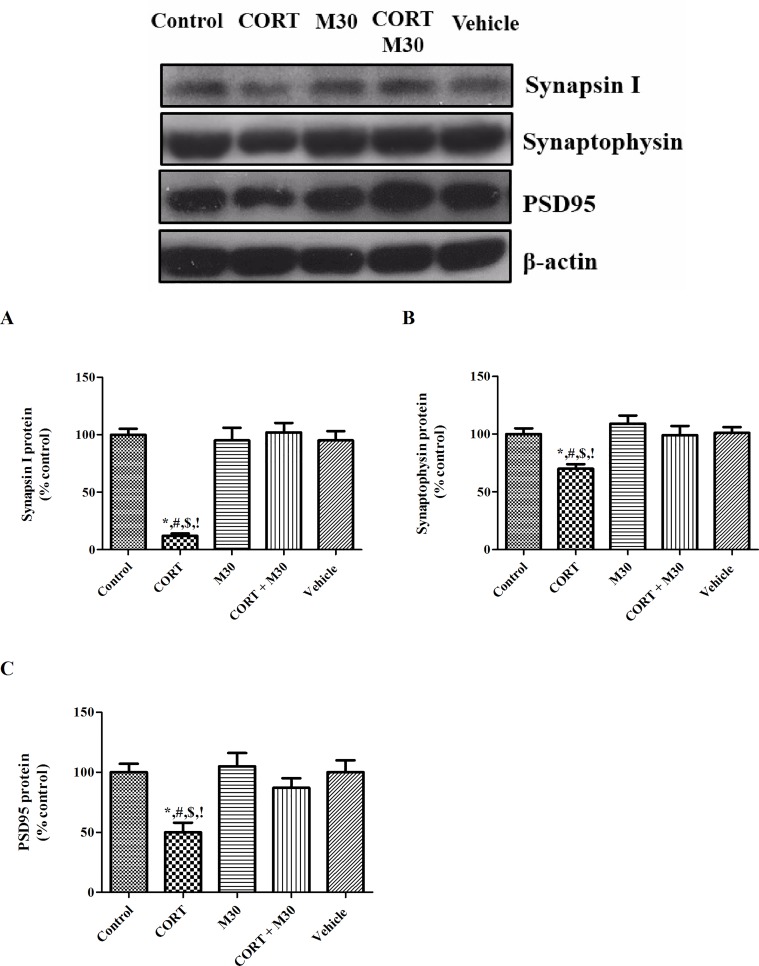
Chronic CORT treatment induced loss of synaptic proteins in the hippocampus, which was significantly restored by M30 pre-treatment. Levels of protein expression of (A) Synapsin-1, (B) Synaptophysin and (C) PSD 95 in the hippocampus of the control, CORT-treated (CORT), M30-treated (M30), CORT and M30 co-treated (CORT+M30) or vehicle groups are summarized. β-actin was an internal control. For synapsin-1, *p < 0.001 when compared with Control, ^#^p < 0.001 when compared with M30, ^$^p < 0.001 when compared with CORT + M30 groups,^!^ p < 0.001 when compared with Vehicle. For synaptophysin, *p < 0.01 when compared with Control, ^#^p < 0.001 when compared with M30, ^$^p < 0.05 when compared with CORT + M30 groups,^!^ p < 0.01 when compared with Vehicle. For PSD-95, *p < 0.01 when compared with Control, ^#^p < 0.01 when compared with M30, ^$^p < 0.05 when compared with CORT + M30 groups,^!^ p < 0.01 when compared with Vehicle.

There were significant decreases in the pre-synaptic vesicle proteins synapsin-1 and synaptophysin, and post-synaptic protein PSD-95, respectively by 90%, 30% and 50% in the CORT-treated group when compared with those of the control or vehicle group (p<0.001 for synapsin-1, p<0.01 for synaptophysin and PSD-95) (n = 8, Fig 7). The decreased expressions were significantly mitigated by the M30 treatment (Fig 7).

### M30 prevented CORT-induced neurodegeneration

**Fig 8:**
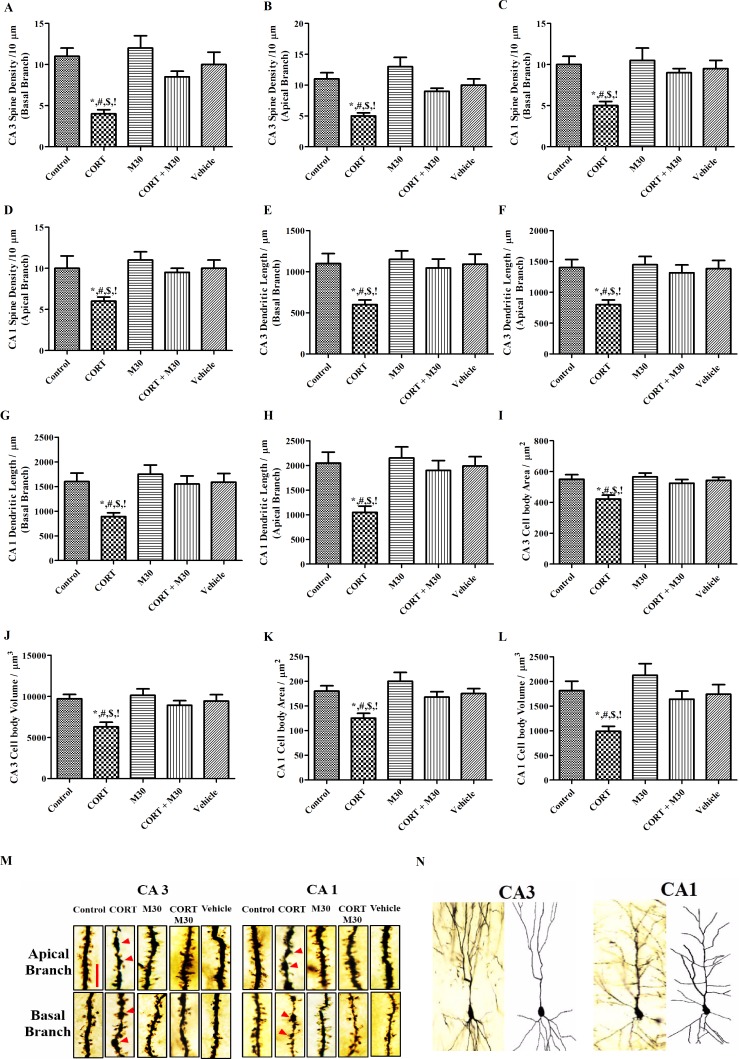
Chronic CORT treatment induced remarkable reductions in dendritic spine density, dendritic length, soma size and volume of both apical and basal branches of CA3 and CA1 pyramidal neurons. M30 administration prevented the aberrant morphological alteration. Dendritic varicosity formations responsible for reduction in dendritic spine density were indicated by the arrows. Dendritic spine densities of apical and basal CA3 and CA1 pyramidal neurons were respectively summarized in Panels A to D. Dendritic length of apical and basal CA3 and CA1 pyramidal neurons were respectively summarized in Panels E to H. Cell bodies’ areas and volumes of apical and basal CA3 and CA1 pyramidal neurons were respectively summarized in Panels I to L. Panel M summarized representative photomicrographs of apical and basal dendrites of both CA3 and CA1 pyramidal neurons showing dendritic spines. Magnification:100X, Scale bar = 10μm. Panel N illustrated Camera Lucida drawing of representative CA3 and CA1 pyramidal neurons. Data from each group were expressed as mean ± SEM (n = 12). Statistical comparisons between groups were performed using the One way Anova followed by Tukey post hoc test to detect differences in all groups. *p < 0.05 when compared with Control, ^#^p < 0.05 when compared with M30, ^$^p < 0.05 when compared with CORT + M30 groups,^!^ p < 0.05 when compared with Vehicle.

The CORT-treated group had significant reductions in the density of dendritic spine, dendritic length, soma size and the volume of both basal and apical branches of CA1 and CA3 pyramidal neurons when compared to the control or vehicle group (p<0.05 for all) (n = 12, Fig 8). There were significant formations of varicosity on the CORT-treated apical and basal dendrites, which were indicated by the arrows in the Fig 8. However, these reductions and formation of varicosity were not observed in the group co-treated with M30 (Fig 8).

## Discussion

It is the first report on the neuroprotective mechanism of M30 against depressive-like behavior resulting from altered serotonin metabolism and depletion of serotonin availability, loss of synaptic proteins and disrupted dendritic neuroarchitecture induced by chronic exposure to CORT. Importantly, we have demonstrated that M30 effectively prevented the pathophysiological consequence upon exaggerated MAO activation, namely oxidative stress and neuroinflammation, which significantly contributes to IDO-1 activation, serotonin deficiency and neurodegeneration. In fact, chronic CORT treatment induced significant depressive-like symptoms accompanied by serotonin deficiency and damaged neuronal structure in the hippocampus, the major brain region responsible for the negative feedback loop of hypercortisolemia. In our study, we have performed forced swimming test and reward-based sucrose preference test to assess behavioral despair and anhedonia, the main phenotypes of depression. Our results demonstrated that there were significant increases in immobility time and decreases in the percentage of sucrose consumption in the CORT-treated group (Fig 1 Panel A and B). These observations are in consistent with previous reports showing the depressive behavior induced by CORT treatment in the rat [5, 31]. In addition, it has been reported that a high dose (50 mg/kg) of CORT is required for the induction of depressive-like behaviors in a 2-week experimental paradigm [5]. This paradigm could consistently induce the pathophysiological cascade leading to neurodegeneration and also it could minimize the confounding side effects of CORT administration over months and the suffering of repeated CORT injections and the number of animals used for the study because of the increased variability and inconsistency of the end point measurements. Besides, the dose of M30 used in this study has been reportedly without adverse impacts on the physiological functions of healthy animals and no any toxicity effect was observed in animals given chronic treatment of M30 [22, 32, 33]. Particularly, M30 administration at the dose of 5mg/kg, which is reportedly the lowest dose effective for the neuroprotection, does not cause the most probable side effects of MAO inhibitors, namely the cheese effects, because M30 is a brain-selective MAO-inhibitor and poorly effects on small intestinal MAO-A activities [34].

We found that the antagonistic effect of M30 on the MAO activity is crucial to the protective action against CORT-induced depression. MAO-A plays a primary role in the maintenance of homeostatic levels of monoamines namely serotonin, norepinphrine and dopamine in the brain. Under pathophysiological conditions, persistent over-activation of the MAO-A activity could result in excessive turnover of monoamines leading to imbalance and deficiency, which significantly contributes to the induction of depressive symptoms. Indeed, we found that there was a significant increase in the MAO-A activity in the CORT-treated group, resulting in elevated serotonin catabolism and lowered serotonin levels in the hippocampus, which were markedly prevented by the co-treatment of M30. This finding is consistent with previous reports that abnormal activation of MAO-A and augmented serotonin turnover were present in the brain of depressed patients and stress-induced depression in animals, in which the depressive symptoms could be ameliorated by MAO-A inhibitors [35–37]. Intriguingly, no observable changes in the levels of 5-HT and 5-HIAA in the M30-treated group suggesting the activation of intrinsic cellular compensatory mechanism to prevent the accumulation of 5-HT leading to toxicity. In addition to be degraded by MAO-A, 5-HT can also be converted to N-acetyltransferase or so called arylalkylamine N-acetyltransferase (AANAT). AANAT has been demonstrated to present in the rat brain, particularly prominent in pineal gland, hippocampus, olfactory bulb, cerebellum, and spinal cord [38]. The levels of 5-HT and 5-HIAA depends on the balance between 5-HT accumulation due to MAO-A inhibition and activation of intrinsic cellular compensatory response

In addition to the deregulated serotonin turnover, over-activation of MAO-A induced by chronic CORT treatment disrupts redox balance as a result of the production of hydrogen peroxide as an enzymatic by-product of catalytic deamination of monoamines. This could significantly increase the production of reactive oxygen species (ROS) that oxidize the lipid membrane forming the MDA, and depleting endogenous antioxidant GSH capacity. In fact, our results showed that chronic CORT treatment significantly increased the MDA level accompanied by decreases in the GSH to GSSG ratio and the protein expression of antioxidant enzymes SOD-2 and GPx-1 in the hippocampus. Remarkably, these aberrant changes could be neutralized by the concurrent administration of M30, highlighting the antioxidant property of M30 against oxidative stress in addition to the MAO inhibition. These results are eventually in line with previous reports demonstrating M30 treatment can ameliorate oxidative stress in in-vivo studies by restoring antioxidant capacity and chelating excessive free radicals [39, 40].

We and others had shown that neuroinflammation was tightly associated with oxidative stress [26, 41]. Oxidative damage of the tissue could lead to the robust release of inflammatory cytokines through the activation of redox-sensitive NFκB canonical pathway. Additionally, these inflammatory mediators exacerbated the positive feedback loop among oxidative stress, inflammation and the tissue damages. Our results showed NFκB canonical pathway was activated through the degradation of the inhibitory protein IκBα leading to the translocation of NFκB members which induced their downstream inflammatory candidates such as TNF-α, IL-1β, IL-6 and COX-2 in the CORT-treated group, but these were potently repressed by M30 treatment. Overwhelming production of hydrogen peroxide is formed as a result of elevated MAO-A activities that serve as an important source of free radicals leading to the development vicious cycle of oxidative stress, neuroinflammation and cellular injuries. The anti-inflammatory ability of M30 demonstrated is possibly attributed to the blockade of vicious cycle through MAO-A inhibition as MAO-A is an early target gene up-regulated by CORT and induce significant oxidative stress. In addition, the anti-inflammatory activity of M30 is also conferred by propargylamine moiety that is the same seen in rasagiline (Azilect) and Ladostigil which have been shown pronounced anti-inflammatory ability [42, 43]. Importantly, these inflammatory mediators triggered cytokine-sensitive IDO-1, the catabolic enzyme of serotonin and tryptophan which is the precursor of serotonin biosynthesis [44, 45]. The elevated IDO-1 activity could increase the conversion of tryptophan into kynurenine, which could decrease the tryptophan availability for serotonin synthesis resulting in serotonin deficiency. This is supported by our results showing significant increases in IDO-1 expression and activity along with a remarkable decrease in serotonin level in the hippocampus of CORT-treated group. These results are in agreement with the previous report which the activation of IDO-1 leading to serotonin deficiency involved in the progression of depressive-like behavior, which could be hindered by application of anti-inflammatory agent [46, 47]. Significantly, the administration of M30 mitigated the CORT-induced neuroinflammation, which is a result of the lowered MAO activity and oxidative stress targeted by M30. In addition to IDO-1, TDO-2 is another enzyme mediating tryptophan metabolism. However, TDO-2 is hardly detectable in the hippocampus of rat and responsive to elevated levels of inflammatory cytokines [48]. In our study, we have shown that there were significant elevations in the inflammatory cytokines which is pivotal to the activation of IDO-1 which subsequently reduced the level of 5-HT in the hippocampus. Our observation is in agreement with the previous reports demonstrating the activation of IDO-1 accompanied by the reduction in the 5-HT content in response to immune activation in the hippocampus of rat [49, 50]. Therefore, it should be IDO-1 instead of TDO-2 mediating the tryptophan breakdown in the hippocampus.

Apoptosis significantly contributes to neuroarchitectural changes that observed in clinically depressed patients and in animals [51, 52]. Our previous studies have shown that oxidative stress and inflammation could lead to neuronal apoptosis through the activation of intrinsic and extrinsic apoptotic cascades [26, 53]. Consistently, oxidative stress and inflammation induced by chronic CORT treatment trigger neuronal apoptosis evidenced by elevated levels of apoptotic markers cleaved caspase-3 and cleaved PARP-1 along with significant decreases in the anti-apoptotic protein Bcl-2. The apoptosis was significantly abrogated by M30 treatment, suggesting that blockade of MAO over-activity, oxidative stress and neuroinflammation are mechanistically involved in the anti-apoptotic effect of M30. Also, our results give support to previous findings showing anti-apoptotic effect of M30 in the brain of animals treated with dexamethasone or neurotoxin MPTP [22, 54].

Synaptic degeneration is suggested to be closely connected to the pathogenesis of depression, resulting from a significant loss of pre- and post-synaptic proteins [55, 56]. Pre-synaptic vesicle proteins play a role in the release of neurotransmitter into the synaptic cleft facilitating the neuronal communication while post-synaptic proteins orchestra the received pre-synaptic signal that in turn to adjust and fine-tune the activity of the dendritic spine [57, 58]. We found that chronic CORT treatment markedly down-regulated the pre-synaptic vesicle proteins synapsin-1 and synaptophysin and the post-synaptic protein PSD-95. These results are in agreement with previous reports showing that chronic CORT treatment could induce significant losses of synaptic proteins [5, 6], which are mediated by apoptotic caspase enzymes [59]. The fact that M30 significantly prevents the loss of synaptic proteins, strongly suggesting that a pathophysiological cascade mediated by MAO over-activity, oxidative stress, neuroinflammation and apoptosis, impeding the loss of synaptic proteins induced by CORT.

Moreover, we found that there were reduced dendritic length and spine density in both apical and basal branches of the CA3 and CA1 pyramidal neurons upon the CORT treatment. It was reported that NMDA excitotoxicity induced by CORT exposure causes the formation of varicosities in the dendritic spine and reduces the spine density [60]. In fact, quinolinic acid, an end product of breaking down tryptophan by IDO-1, is a ligand of NMDA receptors and possibly plays a role in the varicosity formation as a result of the elevated IDO-1 activity upon the CORT treatment [61]. This is supported by the fact that IDO-1 expression and activity, and the level of QUIN were significantly attenuated by the M30 treatment. Consequently, M30 could prevent the varicosity formation and preserve the reduced spine density induced by CORT. Our findings are in agreement with previous observations that impaired dendritic plasticity induced by chronic stress could be rescued by MAO inhibitor [62]. Furthermore, soma size and volume were decreased by the CORT treatment, which could be attributed to the pathogenic processes of neuronal apoptosis when DNA is fragmented and chromatin are condensed [63]. This could explain the protective effect of M30 treatment on the decreased soma size and volume shown in our results. Hence, multifunctional properties of M30 are important to prevent CORT-induced depression and the mechanistic effects of M30 against depressive-like behavior are summarized in the S2 Fig.

## Conclusion

We have demonstrated the neuroprotective mechanism of selective brain MAO inhibitor M30 against CORT-induced depressive behavior by preventing altered serotonin metabolism mediated by elevated MAO-A and cytokine-sensitive IDO-1 activities, and by antagonizing the loss of synaptic protein and impaired dendritic morphologies mediated by oxidative stress, inflammation and apoptosis. Importantly, these works suggest that overactivities of MAO-A and IDO-1 play an important pathophysiological role in the CORT-induced depression, which can be effectively counteracted by M30.

## Supporting Information

S1 Table Chronic CORT treatment induced hypercortisolemia in the rats. Data from each group were expressed as mean ± SEM (n = 12). Statistical comparisons between groups were performed using the One way Anova followed by Tukey post hoc test to detect differences in all groups. *p < 0.001 when compared with Control, ^#^p < 0.001 when compared with Vehicle group.
(PDF)

S1 Fig Chronic CORT treatment elevated protein expression levels of MAO-A and MAO-B, which were markedly attenuated by the M30 treatment. Protein expression levels of (A) MAO-A and (B) MAO-B in the hippocampus of the normoxic (Nx), CORT-treated (CORT), M30-treated (M30), CORT and M30 co-treated (CORT+M30) or vehicle groups are summarized in the figures. β-actin was an internal control. Data from each group were expressed as mean ± SEM (n = 8). Statistical comparisons between groups were performed using the One way Anova followed by Tukey post hoc test to detect differences in all groups. For MAO-A and MAO-B protein expressions, *p < 0.001 when compared with Control, ^#^p < 0.001 when compared with M30, ^$^p < 0.001 when compared with Vehicle groups,! p < 0.001 when compared with Control, ^^^p < 0.001 when compared with M30, ^%^p < 0.001 when compared with Vehicle groups. For MAO-B activity, *p < 0.001 when compared with Control, ^#^p < 0.001 when compared with M30, ^$^p < 0.001 when compared with CORT + M30 groups,^!^ p < 0.001 when compared with Vehicle, ^^^p < 0.001 when compared with Control, ^%^p < 0.001 when compared with CORT + M30, ^&^p < 0.001 when compared with Vehicle groups
(PDF)

S2 Fig Schematic diagram illustrates the neuroprotective mechanism of M30 against depressive like behavior induced by CORT. (PDF)
