# Supplementary material for: M30 Antagonizes Indoleamine 2,3-Dioxygenase Activation and Neurodegeneration Induced by Corticosterone in the Hippocampus
Source: PLoS One. 2016 Nov 21;11(11):e0166966. doi: 10.1371/journal.pone.0166966 (PMC5117770; doi:10.1371/journal.pone.0166966)
Supplement: S1 Table — Data from each group were expressed as mean ± SEM (n = 12). Statistical comparisons between groups were performed using the One way Anova followed by Tukey post hoc test to detect differences in all groups. *p < 0.001 when compared with Control, #p < 0.001 when compared with Vehicle group. (PDF) [file pone.0166966.s001.pdf]

Table 1: Plasma CORT level was elevated after 14-day CORT treatment			
	**Treatment**		
	Control	50mg/kg	Vehicle
[CORT], ng/ml (peak level)	145 ± 20	3300 ± 400$^{*,\#}$	130 ± 24
