# Supplementary figures and images for: M30 Antagonizes Indoleamine 2,3-Dioxygenase Activation and Neurodegeneration Induced by Corticosterone in the Hippocampus
Source: PLoS One. 2016 Nov 21;11(11):e0166966. doi: 10.1371/journal.pone.0166966 (PMC5117770; doi:10.1371/journal.pone.0166966)

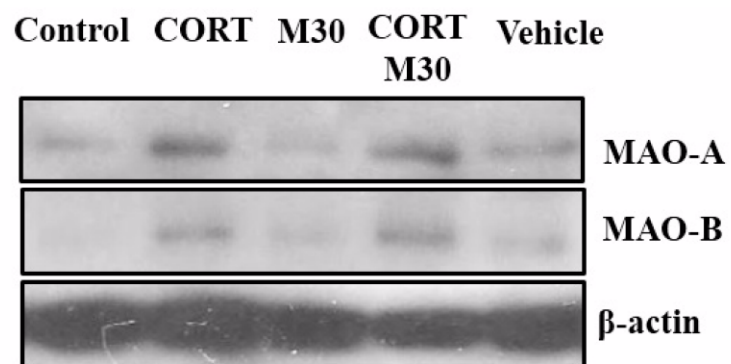

A

B

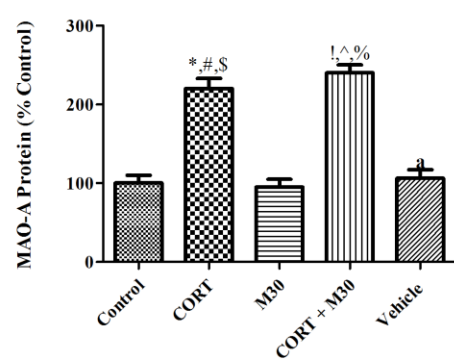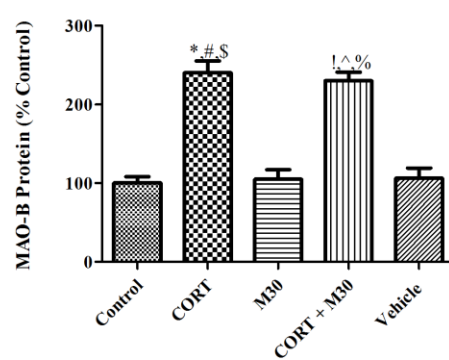

C

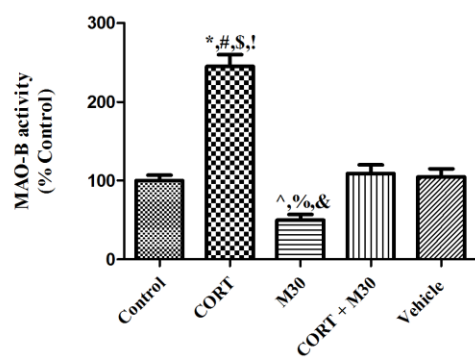

Supplement: S1 Fig — Protein expression levels of (A) MAO-A and (B) MAO-B in the hippocampus of the normoxic (Nx), CORT-treated (CORT), M30-treated (M30), CORT and M30 co-treated (CORT+M30) or vehicle groups are summarized in the figures. β-actin was an internal control. Data from each group were expressed as mean ± SEM (n = 8). Statistical comparisons between groups were performed using the One way Anova followed by Tukey post hoc test to detect differences in all groups. For MAO-A and MAO-B protein expressions, *p < 0.001 when compared with Control, #p < 0.001 when compared with M30, $p < 0.001 when compared with Vehicle groups,! p < 0.001 when compared with Control, ^p < 0.001 when compared with M30, %p < 0.001 when compared with Vehicle groups. For MAO-B activity, *p < 0.001 when compared with Control, #p < 0.001 when compared with M30, $p < 0.001 when compared with CORT + M30 groups,! p < 0.001 when compared with Vehicle, ^p < 0.001 when compared with Control, %p < 0.001 when compared with CORT + M30, &p < 0.001 when compared with Vehicle groups (PDF) [file pone.0166966.s002.pdf]

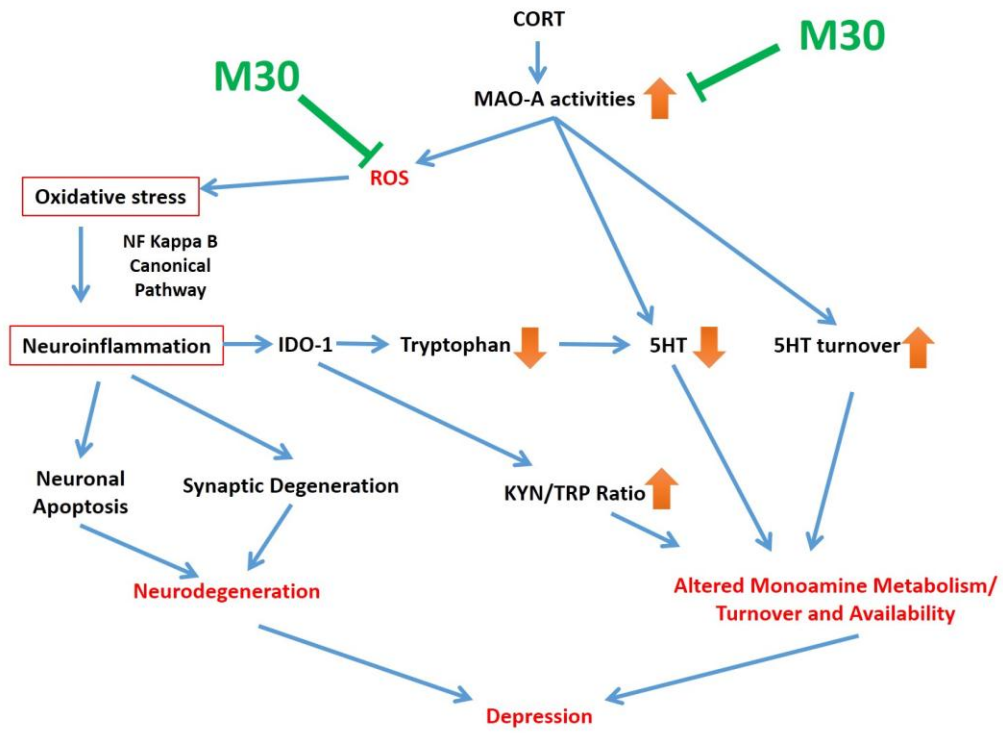

Supplement: S2 Fig — (PDF) [file pone.0166966.s003.pdf]
